# Genome sequences of 13 *Bacillus anthracis* Sterne bacteriophages isolated from soil in the western *United States*

**DOI:** 10.1128/mra.00207-23

**Published:** 2023-11-30

**Authors:** Jacob D. Fairholm, Rebecca James, Emily D. Lavering, Benjamin H. Ogilvie, Trever L. Thurgood, Richard A. Robison, Julianne H. Grose

**Affiliations:** 1 Department of Microbiology and Molecular Biology, Brigham Young University, Provo, Utah, USA; Indiana University, Bloomington, Bloomington, Indiana, USA

**Keywords:** *Bacillus anthracis*, bacteriophage, phage, *Wbetavirus*, *Bastillevirinae*

## Abstract

*Bacillus anthracis,* classified as a Tier 1 Select Agent by the Centers for Disease Control and Prevention (CDC), is the causative agent of anthrax in both humans and livestock. Herein, we report the full genome sequences of 13 bacteriophages that infect *B. anthracis* Sterne. These phages are grouped into four clusters and are similar to previously described *Bacillus* phages.

## ANNOUNCEMENT

*Bacillus anthracis* is a gram-positive, facultatively anaerobic bacterium known as the cause of anthrax and, as such, is classified as a CDC Tier 1 Select Agent (1, 2). We present the sequencing and annotation of 13 *B. anthracis* Sterne bacteriophages isolated from soil samples in the western United States in the summer of 2017 (Table 1). Briefly, ~10 g of top soil was incubated with *B. anthracis* Sterne in LB media at 37°C with shaking for 48–72 hours. Enrichment cultures were then centrifuged to pellet bacteria, and the supernatant was incubated with overnight bacterial culture before plating with LB top agar. Individual plaques were isolated and used to infect fresh overnight bacterial cultures. Plaque purification was repeated three times. Phage genomic DNA from high titer lysates (>10^8^ PFU/mL) was isolated using the phage DNA isolation kit from Norgen Biotek (Canada).

**TABLE 1:** Genomic properties of 13 *Bacillus anthracis* Sterne bacteriophages isolated from soil in the western *United States*

Phage name	GenBank accession no.	SRA accession no.	Total no. of reads	Fold coverage range (mean)	Length (bp)	GC (%)	City, state	GPS^*b*^	Tax^*a*^
vB_BanH_Abinadi	OK500001	SRR25698415	739,121	400–2,868 (815.7)	158,201	38.7	Provo, UT	40.27872, −111.64418	*B*
vB_BanH_Emiliahah	OK499977	SRR25698414	430,530	389–1,524 (601)	158,566	39.9	Idaho Falls, ID	43.61243, −111.87406	*B*
vB_BanH_JarJar	OK499976	SRR25698411	1,060,903	281–2,813 (1,456.2)	161,711	38	Provo, UT	40.24472, −111.65119	*B*
vB_BanH_McCartney	OK499983	SRR25698410	1,116,538	333–5,011 (1,631.1)	158,334	40.1	Idaho Falls, ID	43.61268, −111.90401	*B*
vB_BanH_RonSwanson	OK499990	SRR25742813	1,020,470	897–2,582 (1,329.5)	161,711	37.9	Provo, UT	40.24687, −111.64930	*B*
vB_BanS_Chewbecca	OK499972	SRR25698409	11,616,624	1,094–4,201 (1,640)	161,151	34.4	Elko, NV	40.84541, −115.77714	*S*
vB_BanS_MrDarsey	OK499987	SRR25698408	1,397,758	439–3,016 (1,667.6)	164,998	34.4	Provo, UT	40.24784, −111.64833	*S*
vB_BanS_Nate	OK499992	SRR25698407	44,628	20–168 (61.3)	166,879	33.7	Lamoille, NV	40.69160, −115.47420	*S*
vB_BanS_SkyWalker	OK499994	SRR25698406	1,241,514	818–3,424 (1,593.9)	160,928	34.5	Lehi, UT	40.42849, −111.82623	*S*
vB_BanS_Sophrita	OK499991	SRR25698405	247,586	1–17 (3.8)	167,995	33.9	Provo, UT	40.23906, −111.64995	*S*
vB_BanS_McSteamy	OK499979	SRR25698404	3,380	1–38 (17)	39,748	45.6	Provo, UT	40.26461, −111.64975	*W*
vB_BanS_Booya	OK499999	SRR25698413	11,588	7–114 (62.8)	39,660	35.3	Provo, UT	40.24030, −111.64993	*W*
vB_BanS_Athena	OK500002	SRR25698412	20,090	59–301 (121.4)	37,369	35	Lehi, UT	40.42849, −111.82623	*S*

^*a*^ *Bastillevirinae (B), uncharacterized Siphoviruses (S),* and *Wbetavirus (W*) as determined by the taxonomy given in the NCBI BLASTn taxonomy tab.

^*b*^ Top soil was taken from a wide variety of environments including the BYU campus, local parks, and flower and vegetable gardens at homes.

Genomic DNA was prepared for 250-bp Illumina HiSeq 2500 paired-end sequencing using the Illumina TruSeq DNA Nano kit (see Table 1 for a summary). Trimming of reads and *de novo* assembly were performed using Geneious v.R11 (3) followed by annotation using DNA Master 5.23.6 (4) and GeneMarkS 4.28 (5) with BLASTp (6) used to assign functions. All programs (including Geneious-based trimming) were used at default settings.

Dot plot analysis using Gepard 1.30 (7) showed these phages clustering into four distinct clusters after which the closest relatives and taxonomy were identified using the web-based NCBI BLASTn (September 2023) (6). All genomes circularized and were approximately of the same size as related phages in GenBank. Base pair 1 of all 13 phages was called by alignment with relatives. Cluster one (Abinadi, Emiliahah, McCartney, JarJar, and RonSwanson) comprises members of the subfamily *Bastillevirinae*. All five phages contain a type 1 thymidylate synthase and a dihydrofolate reductase characteristic of this family (8). Abinadi’s closest relative is the *Wphvirus* DIGNKC (accession number: KU737349) with >95% nucleotide identity over >96% of the genome. Emiliahah and McCartney both join the genus *Tsarbombavirus* with >93% nucleotide identity over >89% of the genomes to phage QCM8 (KX961630). Finally, JarJar and RonSwanson join the genus *Bequatrovirus* with their closest relative being Phrodo (KU737347) with >98% identity over >98% of the genome. Cluster two (Chewbecca, MrDarsey, Nate, SkyWalker, and Sophrita) join a group of siphoviruses that currently have no NCBI taxonomic assignment. Izhevsk (MT254578) was the closest relative to Chewbecca, MrDarsey, and SkyWalker with >94% identity over >87% of the genome, pW2 (MK288021) was Sophrita’s closest relative with >93% nucleotide identity over 78% of the genome, and Nate’s was Thrax5 (ON548421) with >89% over 83% of the genome. Cluster three (McSteamy and Booya) joins the *Wbetavirus* genus. The closest relative to McSteamy is J5a (MT745955) with >92% nucleotide identity over 79% of the genome, while Booya has >94% nucleotide identity over 76% of phage AP631 (MK085976). Athena has only distant phage relatives [such as BVE2 (MG584725), sharing 93% nucleotide identity over only 8% of the genome] but encodes an integrase and has 100% sequence identity to *Bacillus anthracis* genomes (including strain FDAARGOS) consistent with temperate phages.

## Data Availability

See Table 1 for NCBI accession numbers.
